# An evaluation of a volumetric method for the flow cytometric determination of residual leukocytes in blood transfusion units

**DOI:** 10.1371/journal.pone.0279244

**Published:** 2022-12-19

**Authors:** Elvira Maličev, Klara Železnik, Katerina Jazbec

**Affiliations:** 1 Department of Immunohematology, Blood Transfusion Centre of Slovenia, Ljubljana, Slovenia; 2 Biotechnical Faculty, University of Ljubljana, Ljubljana, Slovenia; The Ohio State University, UNITED STATES

## Abstract

The removal of leukocytes from blood components helps to prevent or reduce some adverse reactions that occur after blood transfusions. The implementation of the leukodepletion process in the preparation of blood units requires quality control, consisting of a reliable cell counting method to determine residual leukocytes in blood components. The most widely used methodology is a flow cytometric bead-based counting method. To avoid the need for commercial counting beads, we evaluated a volumetric counting method of leukocyte enumeration. A total of 160 specimens of leukodepleted plasma, red cell and platelet units, as well as 58 samples of commercially available controls containing different concentration levels of leukocytes, were included in the study. The conventional quality control method using the bead-based counting method performed with the FACSCalibur flow cytometer was compared to the bead-based counting method and the volumetric counting method performed with the MACSQuant 10 flow cytometer. Our results show that the MACSQuant bead-based method, as well as the volumetric MACSQuant method, meet the sensitivity requirements of residual leukocyte enumeration when compared to the gold standard, bead-based FACSCalibur method. We conclude that the volumetric method can be a substitute for the bead-based counting of residual leukocytes in a variety of blood components.

## Introduction

Whole blood components are now rarely used for transfusion. After blood collection, whole blood is separated into blood components and stored as red cell components, platelet components, and fresh frozen plasma. This practice allows each component to be stored at its optimal temperature and enables the administration of specific replacement therapies to patients.

Blood components can be effectively separated from whole blood by differential centrifugation or by apheresis. Leukocyte-reduction filters are used to remove residual leukocytes from blood components derived from whole blood to decrease the potential risk of cell-mediated adverse reactions after the transfusion. The presence of leukocytes in blood components can cause febrile transfusion reactions [1, 2], alloimmunization to foreign antigens [3, 4], and the transmission of viruses [5, 6]. The process of leukodepletion from a blood product before transfusion may also decrease the probability of bacterial contamination of blood components [7, 8]. For these reasons, transfusion centers perform leukodepletion for the majority of blood components. To ensure their best possible safety, quality control measurements of residual leukocytes after the leukodepletion are regularly carried out. According to the guidelines of the European Committee leukodepleted blood products are required to have residual leukocytes below 1 × 10^6^ leukocytes per blood unit in more than 90% of the tested cellular and plasma units, while non-leukodepleted plasma is required to have < 0.1x 10^9^ leukocytes per liter [9].

In blood components, counting low levels of residual leukocytes with high degree of precision is challenging. Since leukocyte concentrations in blood products are below the level of their ability to detect them, most automated hematology analyzers are therefore not suitable for this use. Therefore, the residual leukocyte count in a small sample volume is determined with other methodologies, including microscopy and flow cytometry. The manual microscopy with a Nageotte chamber, which was one of the first residual leukocyte counting methods, or a portable fluorescent microscopic leukocyte cell counter ADAM-rWBC (NanoEntek), are two well-known non-flow cytometric counting procedures [10–12]. As both have their limitations, the Nageotte hemocytometer is less accurate and less precise and is time-consuming, the ADAM-rWBC shows better results for low-level leukocyte enumeration in blood samples, but still has a narrow scope [13, 14], flow cytometry is a much more flexible technique and can achieve counts even when there are very low cell numbers. Currently, the use of a flow cytometer with commercial reagents for cell staining is the most widely used technique for low-level leukocyte enumeration [15, 16].

Since the volume of a sample passing through the flow cytometer during data acquisition is not known, most flow cytometers do not calculate the absolute number of cells in the samples directly. The absolute cell number can be exactly calculated indirectly using a known number of commercial beads added to the cell sample. With the recent development of some newer types of flow cytometers, which can measure the exact volume of analyzed cell suspension, the bead-based counting method can be avoided. Flow cytometers that allow a volumetric determination of cell counts in the blood samples are MACSQuant 10 (Miltenyi Biotec), BD Accuri C6 (BD), Guava easyCyte and Attune Nxt (Thermo Fisher Scientific), and CyFlow (Sysmex). In routine hematological laboratory testing, such flow cytometers are rare and therefore the bead-based leukocyte enumeration technique is the most frequently used for the quality control of blood units.

In our institution, the Blood Transfusion Centre (BTC) of Slovenia, residual leukocytes in the red cell, platelet, and plasma units are determined using the flow cytometer BD FACSCalibur (BD) and commercially available reference counting beads. About 1500 units are analyzed annually by the department of our institution which deals with the final stage of quality control. In our study, we performed comparative analyses of different residual leukocyte enumeration results using the flow cytometers FACSCalibur and MACSQuant 10. As MACSQuant 10 allows volumetric measurements, we also evaluated the performance of volumetric enumeration of low levels of leukocytes in leukodepleted blood units. We show that the results were comparable and that the need for expensive counting beads could be avoided.

## Methods

### Study design

A total of 218 samples were analyzed on the flow cytometer BD FACSCalibur (BD Biosciences, San Jose, CA, USA), where the leukocyte count was determined with the bead-based counting method. The same samples were analyzed with the flow cytometer MACSQuant 10 (Miltenyi Biotec, Bergisch Gladbach, Germany), where the leukocyte count was determined with the bead-based counting method and with the volumetric counting method.

Commercially available control cells containing different concentration levels of leukocytes (n = 58), and different blood unit samples (n = 160) were included in the study. Two concentration levels of PLT control cells, low and high, and two concentration levels of RBC control cells, also low and high, were used. The PLT control cells are used to control the enumeration of residual leukocytes in leukoreduced platelet units. The RBC control cells are used to control the enumeration of residual leukocytes in leukoreduced red blood cell products. Both types of control cells were shipped from R&D systems EUROCELL Diagnostics. Additionally, the third set of control cells, used only for the study, were prepared by mixing low and high concentration levels of control cells in a ratio of 1:1.

Donor unit samples were taken from different types of red cell, platelet, and plasma components. All components were produced in the usual fashion in our blood transfusion centre. Leukocyte-depleted erythrocytes were prepared from whole blood and processed with special filters that remove ≥ 99.99% of leukocytes (CompoFlex^®^ Quadruple System Fresenius Kabi; top and top or top and bottom system). Platelet components were recovered from whole blood (TERUMO BCT TACSI PL) or obtained by apheresis (AMICUS Apheresis Kit–Single Needle, Fresenius Kabi AG). Plasma was prepared from whole blood or by apheresis or trombopheresis. Plasma samples for flow cytometric analysis were taken before freezing.

### Determination of residual leukocytes by counting beads

Donor samples, PLT and RBC controls were prepared using BD Leucocount Kit (BD Bioscience, San Jose, CA, USA), according to the manufacturer´s instructions. Briefly, 100 μL of well-mixed cell sample was dispensed into a Trucount tube (BD Bioscience, San Jose, CA, USA), which contained a predefined number of counting beads. Cells were labeled by adding 400 μL of Leucocount reagent to each tube. The reagent contained a nucleic acid dye propidium iodide (PI) and RNAse, and therefore only cellular DNA was stained. The tubes were gently mixed and incubated for 5 min in the dark at room temperature. All PLT and RBC control cells were treated in the same way as specimen samples.

After staining, samples were analyzed with the FACSCalibur (BD Biosciences, San Jose, CA, USA), using the CellQuest Pro software and a BD template for residual leukocytes enumeration, and the MACSQuant 10 (Miltenyi Biotec, Bergisch Gladbach, Germany), using the MACSQuantify software (Miltenyi Biotec, Bergisch Gladbach, Germany) and a modified in-house template (time/leukocytes and time/beads, see S1 Fig). Ten thousand events of the bead population were recorded at low flow speed and analyzed.

The absolute leukocyte count was determined using the following formula:

leukocytes/μl=totalbeads×leukocytesacquired/beadsacquired×samplevolume

### Determination of residual leukocytes by volumetric model

The MACSQuant is equipped with a precise fluidics system that enables the automated and accurate uptake of sample volumes, i.e., volumetric pipetting. The MACSQuantify program automatically calculates the events/μl in the leukocyte (WBC) gate. The given result was multiplied by 5, as the original sample was diluted with PI staining solution (samples were run sequentially, after the routine analysis on the FACSCalibur). Approx. 160 μl of diluted sample was recorded at low flow speed.

### Statistical analysis

#### Intra-laboratory reproducibility

To evaluate precision, a total of 58 runs of PLT and RBC controls were carried out on both systems. To assess the precision of each method, we calculated the mean values, standard deviations (SD), and intra-method coefficients of variation (CVs).

#### Method comparison

Flow cytometric results of the PLT and RBC controls (n = 51) measured on FACSCalibur and MACSQuant 10 were analyzed for the absolute leukocyte count. To obtain data on cell concentration between the low and the high concentration of leukocytes, some PLT and RBC control samples were also mixed in a 1:1 ratio before staining (n = 7).

To evaluate a bias between the mean differences of measurements made with the MACSQuant and the FACSCalibur systems, a Bland-Altman analysis was performed using IBM SPSS Statistics 28 (PS IMAGO PRO 8.0, Krakow, Poland). Regression analysis was also performed, and Pearson’s correlation coefficient was calculated.

## Results

### Precision and accuracy of leukocyte determination by flow cytometers

The precision of both methods was tested using stable platelet (PLT) and red blood cell (RBC) controls with low and high leukocyte concentrations. Each sample was prepared and run 7 times on different days on both systems. The mean value of leukocytes per microliter in cell controls measured with the bead-based counting method and the volumetric method, and acquired using the two flow cytometer systems, FACSCalibur and MACSQuant 10, are shown in Table 1. To assess the precision of each method, we calculated the intra-method coefficients of variation (CV).

**Table 1:** Flow cytometry results of leukocyte counts (WBC/μl) in PLT and RBC controls for two dilution series.

	Expected values (WBC/μl-range)	FACSCalibur bead-based (WBC/μl, n = 7)	MACSQuant bead-based (WBC/μl, n = 7)	MACSQuant volumetric (WBC/μl, n = 7)
**PLT low control**	0.5–3.5			
mean		2.10	2.30	2.21
SD		0.42	0.61	0.59
CV		19.5%	26.6%	26.8%
**PLT high control**	15.7–26.3			
mean		19.77	19.45	19.73
SD		1.66	1.61	1.85
CV		8.4%	8.3%	9.4%
**RBC low control**	0.7–3.7			
mean		2.04	2.51	2.38
SD		0.43	0.31	0.20
CV		21.1%	12.5%	8.6%
**RBC high control**	15.0–25.0			
mean		19.34	20.15	19.00
SD		1.01	0.98	0.87
CV		5.2%	4.9%	4.6%

Mean leukocyte counts (mean), standard deviation (SD), and coefficient of variation (CV) were calculated from the results obtained with the bead-based method on the FACSCalibur and MACSQuant 10, and with the volumetric method on the MACSQuant 10.

On average, CVs were higher in the PLT and RBC control samples with lower leukocyte concentrations. The CVs did not differ much between all three methods, except for the RBC low control measurements, where the CVs for the FACSCalibur bead-based method was slightly higher than for others. All results were in the expected range, which was ≤10% for the high controls and ≤30% for the low controls.

### Method comparison study

For the method comparison study, samples were prepared from low and high concentration levels of PLT control cells and low and high concentration levels of RBC control cells; the third samples were prepared by mixing a low-level sample with a high level of PLT controls in a ratio of 1:1. The same was done with RBC controls. In total, 5 different PLT and 5 different RBC controls were used for preparing a total of 58 control samples. The blood samples contained 0–23 leukocytes per μl.

**Fig 1:**
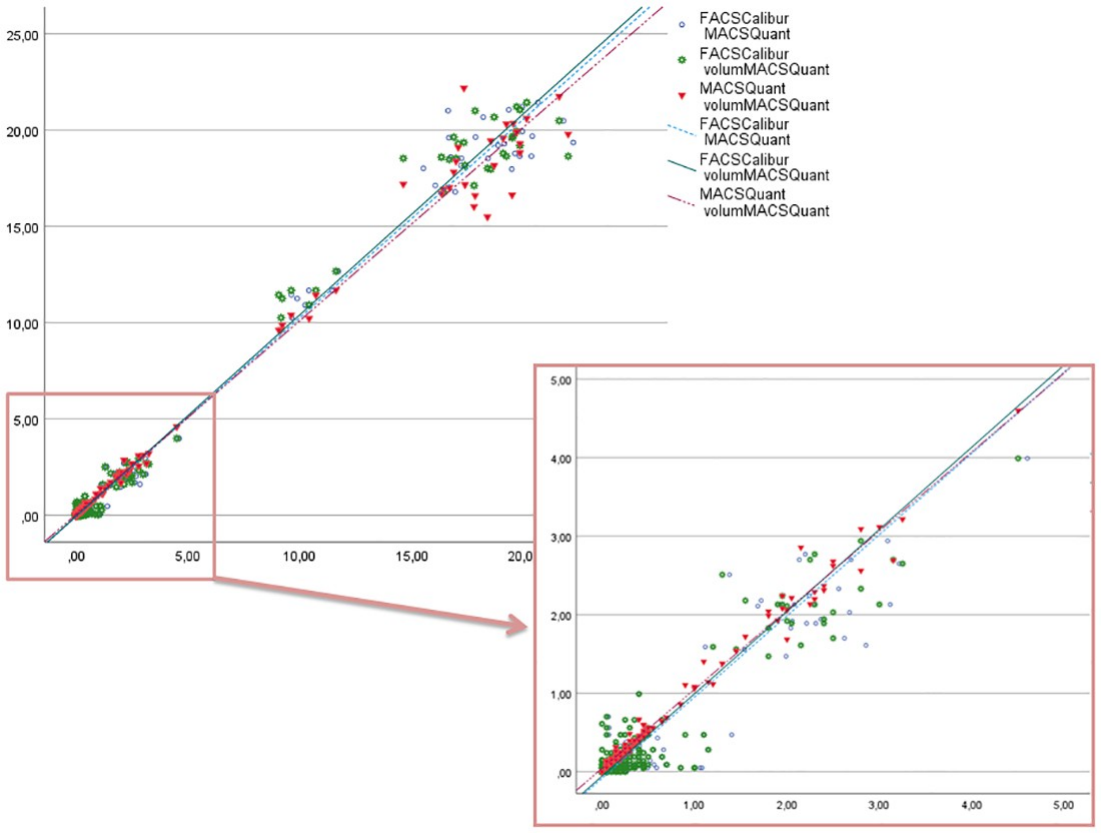
Regression lines. Scatter diagram showing the method comparison between the FACSCalibur bead-based method and the MACSQuant 10 bead-based method (the intercept (95% CI) was -0.085 (-0.183, 0.014), the slope (95% CI) was 1.035 (1.020, 1.050) and R2 was 0.989; *p < 0*.*001*), between the FACSCalibur bead-based method and the MACSQuant 10 volumetric method (the intercept (95% CI) was -0.055 (-0.153, 0.042), the slope (95% CI) was 1.047 (1.032, 1.062) and R2 was 0.989; *p < 0*.*001*), and between the MACSQuant 10 bead-based method and the MACSQuant 10 volumetric method (the intercept (95% CI) was 0.042 (-0.043, 0.127), the slope (95% CI) was 1.007 (0.994, 1.020) and R2 was 0.991; *p < 0*.*001*). The X-axis and Y-axis represent the number of leukocytes per μl.

Samples were analyzed on different days on both flow cytometric systems. In Fig 1, we report the values of leukocyte/μl, measured using all three methods: the bead-based method on FACSCalibur, the bead-based method on the MACSQuant 10, and the volumetric method on the MACSQuant 10.

**Fig 2:**
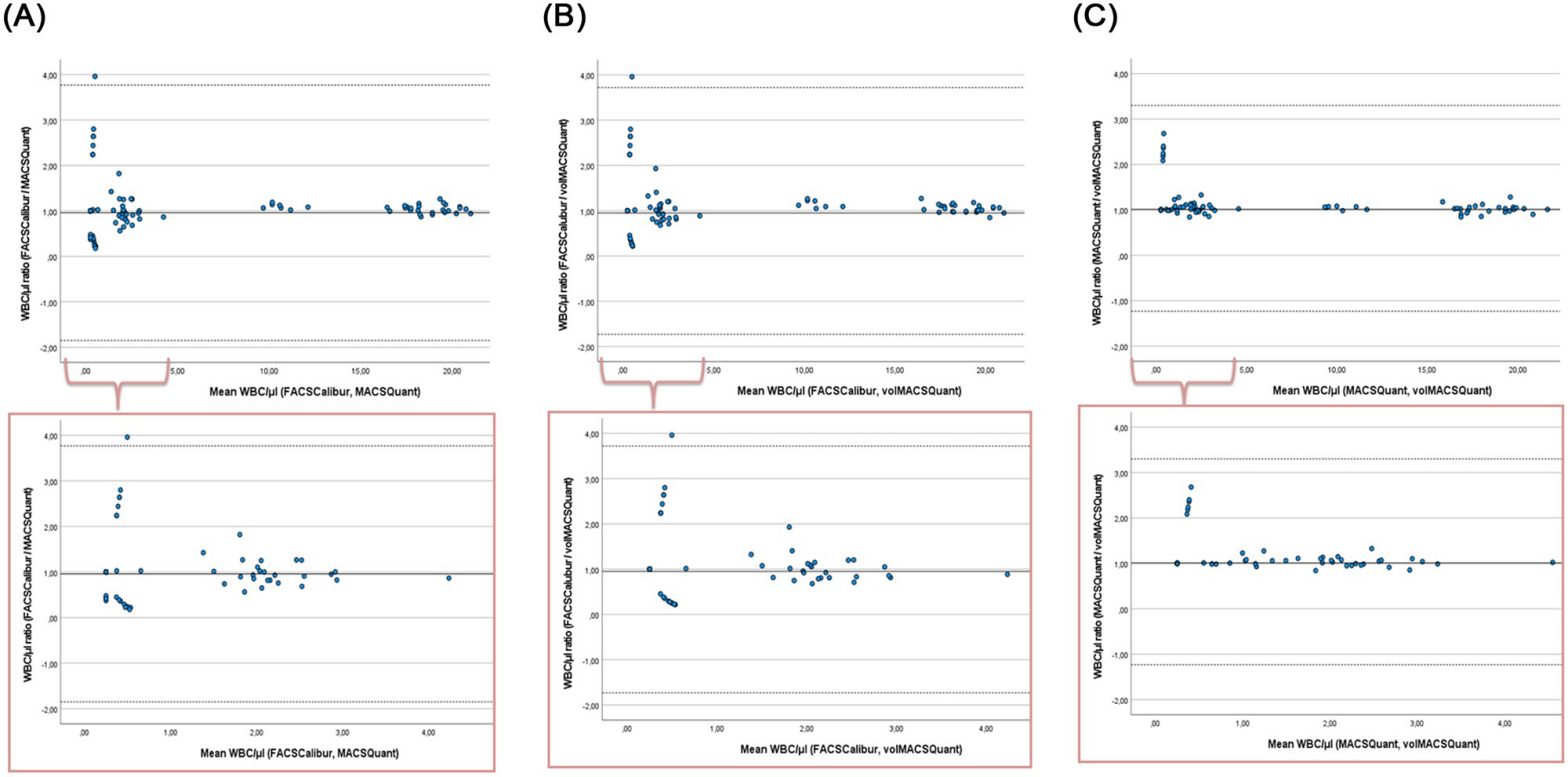
Bland-Altman analysis. Bland-Altman plots show the difference between the two paired measurements plotted against the mean of the two measurements. The events between 0 and 0.5 were considered as 0.25. As the differences were not normally distributed, a logarithmic transformation and back-transformation (antilog) were made. Y-axis: geometric means ratio of the leukocyte count in the controls and samples (n = 218) between the FACSCalibur bead-based method and the MACSQuant 10 bead-based method (A), between the FACSCalibur bead-based and the MACSQuant 10 volumetric method (B), and between the MACSQuant bead-based method and the MACSQuant volumetric method (C). The ratio was 0.96 with 95% limits of agreement -1.85 to 3.77 for A, 0.99 with 95% limits of agreement -1.73 to 3.72 for B, and 1.03 with 95% limits of agreement -1.24 to 3.30 for C. 95% limits of agreement was defined as bias ± 1.96 standard deviation of the difference. X-axis: the mean of WBC/ μl for the same pairs of methods, respectively. Legend: bold line = ratio, doted lines = 95% limits of agreement.

To quantify the agreement between measurements, samples were analyzed with the Bland-Altman method, using 95% limits of the agreement defined as bias ± 1.96 standard deviation (SD) of the difference. Plots of ratios between methods are presented in Fig 2. The data did not have a normal distribution and had to be log-transformed; the events between 0 and 0.5 were considered as 0.25. Back-transformation (antilog) gave the geometric means ratio between the FACSCalibur bead-based method and the MACSQuant bead-based method 0.96 with 95% limits of agreement -1.85 to 3.77, between the FACSCalibur bead-based method and the MACSQuant volumetric method 0.99 with 95% limits of agreement -1.73 to 3.72, and between the MACSQuant 10 bead-based method and the MACSQuant 10 volumetric method 1.03 with 95% limits of agreement -1.24 to 3.30. There was no proportional bias. Our results show the MACSQuant 10 bead-based method, as well as the MACSQuant 10 volumetric method, meet the sensitivity requirements in residual leukocytes enumeration compared to the golden standard of the bead-based FACSCalibur method.

When counting such low numbers of leukocytes, variation is expectedly higher. To see if the system is robust enough to discriminate accurately between zero leukocytes from 3–4 leukocytes per μl, we have also made a series of dilutions in the range of 0, 1, 2, and 4 leukocytes/μl with filtered plasma (prepared from the whole blood with measured 0 leukocytes/μl) spiked with leukocytes from the PLT control cells (high concentration, R&D systems EUROCELL diagnostics). R-squared for all three pairs of methods was above 0.915, p < 0.001 (data not shown). Considering all our data, the MACSQuant 10 bead-based method, as well as the MACSQuant 10 volumetric method, meet the sensitivity requirements in the residual leukocyte enumeration compared to the golden standard of the FACSCalibur bead-based method. The analysis sensitivity requirements are further described in the Discussion.

Our analyzed samples were taken from the blood components with the following volumes (means ± standard deviation): red blood cell units were 293 ± 15 ml (top and top bag) and 264 ± 16 ml (top and bottom bag), platelets recovered from whole blood were 333 ± 22 ml, apheresis platelets 278 ml, plasma recovered from whole blood was 200 ml and apheresis plasma was 500 ml. The number of leukocyte counts with different methods did not exceed the allowed 1 × 10^6^ leukocytes per unit.

## Discussion

The leukodepletion of the blood units, which has been implemented to reduce transfusion-associated risks and has become a standardized practice in the blood banks, requires the measurement and quality control of residual leukocytes. Since the leukocyte concentrations in different types of leukocyte-depleted blood units are below the level of detection for most of the standard hematology analyzers, blood banks use different alternative methodologies to check the residual leukocyte count. Because flow cytometers are versatile, they have also become indispensable laboratory equipment in blood banks and transfusion centers. In transfusion medicine these days, flow cytometry is mainly used for the quality control testing of blood units, the detection of fetomaternal hemorrhage, red blood cell phenotyping in the reference laboratory setting, and the analysis of platelets and granulocyte antigens and their antibodies. As cellular therapies have become a part of transfusion medicine, the use of flow cytometry is now required for hematopoietic stem cell enumeration, mesenchymal stem cell phenotyping, lymphocyte subpopulation detection, and in all other autologous or allogeneic cell transplantation settings. With a flow cytometer’s versatile use, the purchase of special equipment for residual leukocyte counting is not required. Some recent flow cytometers are also equipped to provide the absolute volume of samples analyzed. In these cases, we can determine leukocyte concentration even without the use of commercial counting beads. When blood component leukodepletion became a mandatory requirement around the year 2000, no volumetric cytometers in that period proved reliable for enumerating low numbers of residual leukocytes (Ortho Cytoron Absolute, Partec PAC FCM, IMAGN 2000), for this reason, bead-based absolute counting systems largely prevailed over the decades [17]. With the advancement of technology, we can expect this to change.

Our study compared the bead-based counting method on the FACSCalibur and on the MACSQuant 10 flow cytometers. In parallel, the volumetric method results on the MACSQuant 10, without the use of counting beads, were compared to those of the bead-based FACSCalibur method, which is considered the gold standard [18] and has been used in our BTC facility since 2004. First, we evaluated the reproducibility of the residual leukocyte count with all the three methods. Therefore, the same commercial control cells with low and high leukocyte concentrations were analyzed several times on various days. The intra-method coefficients of variability (CVs) calculated were predictably higher at lower leukocyte values, the highest was in the range of 0.5–3.5 leukocyte/μl in PLT control. However, according to our results collected and presented in Table 1, the count methods do not differ from each other significantly.

We analyzed method comparison data using regression analysis and the Bland-Altman method for the leukocyte range of 1–25 leukocytes/μl. Our results show that in residual leukocyte enumeration, when compared to the bead-based FACSCalibur method, the MACSQuant bead-based method as well as the volumetric MACSQuant method both meet sensitivity requirements. It was previously determined that with the threshold of < 1 × 10^6^ leukocytes per component, borderline unit concentrations are below 3.3 leukocyte/μl [11].

Besides PLT and RBC control samples, specimens of red cell, platelet, and plasma units were analyzed. For all three flow cytometric methods, leukocytes count results were in the range of 0 to 0.99 leukocyte/μl. These are very low numbers, therefore the variation of results was expectedly higher. As this variation was < 3.3 leukocyte/μl, the MACSQuant bead-based method, as well as the volumetric MACSQuant method, still met the sensitivity requirements in residual leukocyte enumeration compared to the gold standard of the bead-based FACSCalibur method.

It is very important to ensure a reliable count due to the extremely low levels of residual leukocytes. At least 160 μl of the diluted sample should be recorded, at low speed, in standard mode (approx. 5–6 min). We recommend using the time gate, and if the time disruption due to the clog is significant, the volumetric counting needs to be repeated. We also recommend setting pre-run gentle mixing, with mixing lower volume than the sample volume, to avoid bubble formation.

We acknowledge the limitations of this study. Our samples were run sequentially after the routine bead-based analysis on the FACSCalibur. Before implementing volumetric counting into routine an external quality assessment scheme for the enumeration of residual leukocytes in leukoreduced blood components, like UKNEQAS, should also be implemented and an inter-laboratory study conducted [19].

Transfusion centers perform leukodepletion on the majority of blood components, as the removal of leukocytes is very important for the health of the recipients. To maximize positive outcomes, quality control measurements are crucial and an adequate robust counting method for residual leukocytes is essential. Our results show that the MACSQuant 10 flow cytometer, using either the bead-based or the volumetric method, is an effective tool in determining the measurements of residual leukocytes in either plasma, platelet, or red cell units. Volumetric counting can be a legitimate alternative, and has the added benefit of reducing costs for the purchase of test tubes and counting beads.

## Supporting information

S1 Fig Gating strategy for bead-based and volumetric method on the MACSQuant 10. (TIF)
