# Supplementary figures and images for: An evaluation of a volumetric method for the flow cytometric determination of residual leukocytes in blood transfusion units
Source: PLoS One. 2022 Dec 19;17(12):e0279244. doi: 10.1371/journal.pone.0279244 (PMC9762584; doi:10.1371/journal.pone.0279244)

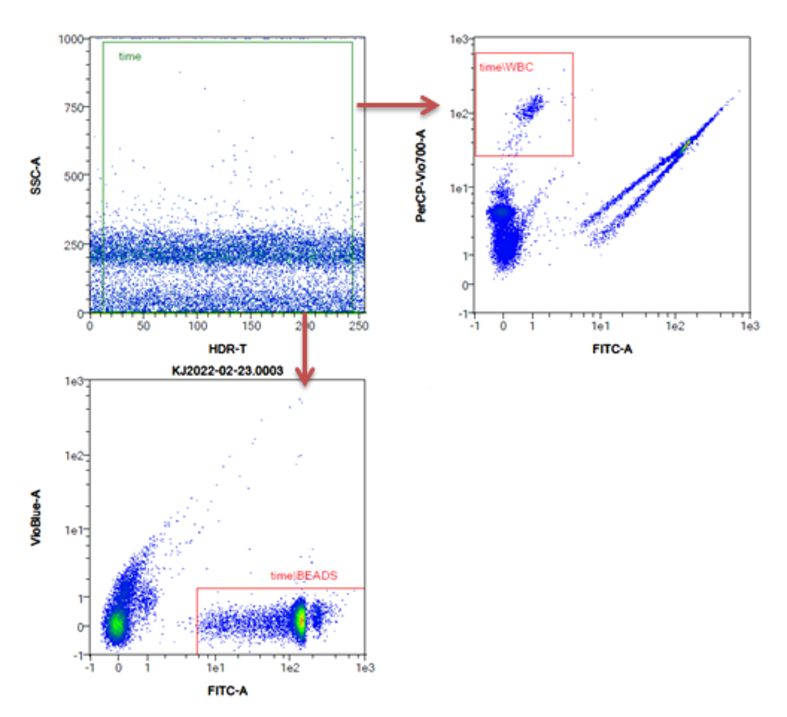

Supplement: S1 Fig — (TIF) [file pone.0279244.s001.tif]
